# An Asp376Glu substitution in *ALS* gene and enhanced metabolism confers high tribenuron-methyl resistance in *Sinapis alba*

**DOI:** 10.3389/fpls.2022.1011596

**Published:** 2022-11-11

**Authors:** Candelario Palma-Bautista, José G. Vázquez-García, Maria D. Osuna, Blanca Garcia-Garcia, Joel Torra, Joao Portugal, Rafael De Prado

**Affiliations:** ^1^ Department of Biochemistry and Molecular Biology, University of Cordoba, Cordoba, Spain; ^2^ Plant Protection Department, Scientific and Technological Research Centre of Extremadura (CICYTEX), Guadajira, Badajoz, Spain; ^3^ Department of Hortofructiculture, Botany and Gardening, Agrotecnio-CERCA Center, University of Lleida, Lleida, Spain; ^4^ Biosciences Department, Polytechnic Institute of Beja, Beja, Portugal

**Keywords:** acetolactate synthase, auxin mimics, chemical control, wheat-weed, cross-resistance

## Abstract

Acetolactate synthase (ALS) inhibiting herbicides (group 2) have been widely applied for the last 20 years to control *Sinapis alba* in cereal crops from southern Spain. In 2008, a tribenuron-methyl (TM) resistant (R) *S. alba* population was first reported in a cereal field in Malaga (southern Spain). In 2018, three suspected R *S. alba* populations (R1, R2 and R3) to TM were collected from three different fields in Granada (southern Spain, 100 km away from Malaga). The present work aims to confirm the putative resistance of these populations to TM and explore their resistance mechanisms. Dose–response assays showed that the R1, R2 and R3 populations ranging between 57.4, 44.4 and 57.1 times more resistance to TM than the susceptible population (S). A mutation in the *ALS* gene (Asp376Glu) was detected in the Rs *S. alba* populations. ^14^C-metabolism studies show that metabolites and TM were changing significantly faster in the R than in the S plants. Alternative chemical control trials showed that 2,4-D and MCPA (auxin mimics), glyphosate (enolpyruvyl shikimate phosphate synthase,EPSPS, inhibitor-group 9), metribuzin (PSII inhibitors/Serine 264 Binders, -group 5) and mesotrione (hydroxyphenyl pyruvate dioxygenase, HPPD, inhibitor-group 27) presented a high control of the four populations of *S. alba* tested, both S and R. Based on these results, it is the first case described where the Asp376Glu mutation and P450-mediated metabolism participates in resistance to TM in *S. alba*. Comparing these results with those found in the *S. alba* population in Malaga in 2008, where the resistance was TSR type (Pro197Ser), we can suggest that despite the geographical proximity (over 100 km), the resistance in these cases was due to different evolutionary events.

## Graphical Abstract

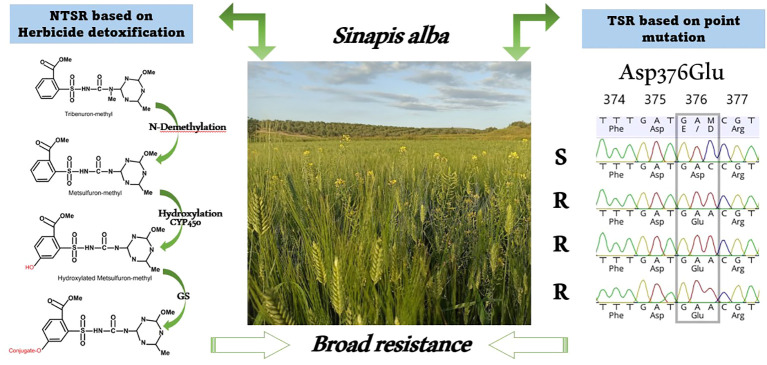

## 1 Introduction

Brassicaceae is a botanic family which contains around of 3,709 species, including oilseed crops, vegetables, and weeds (Warwick et al., 2005). *Sinapis alba* L, also known as white mustard, is a diploid (2x = 2n = 24) wild member of this family. This is a competitive weed in cereal systems in southwest Spain. ALS inhibiting herbicides are frequently used for selective dicotyledonous weed control in these cereal production areas of Spain, increasing over the past 20 years the reports of erratic control of this species with ALS-inhibitors, mainly with tribenuron-methyl (TM) (Rosario et al., 2011; Cruz-Hipolito et al., 2013).

The ALS enzyme (also known as acetohydroxy acid or acetohydroxyacid synthase, abbr. AHAS) participates in the biosynthesis pathway of the branched amino acids, valine, leucine and isoleucine (Shaner, 1991; Duggleby et al., 2008). Nowadays, there are six herbicide families which inhibit this enzyme; the sulfonylureas (SU), imidazolinones (IMI), triazolopyrimidines (TP), pyrimidinyl-thiobenzoates (PTb), sulfonanilides (SA) and triazolinones (SCT) (Heap, 2022). Recently, some of these herbicides have become the main tools to control monocotyledonous and dicotyledonous weeds in various crops (Tranel and Wright, 2022; Nakka et al., 2017; Rigon et al., 2020). Resistant weed populations appear when the same herbicides are used several times a year or several years. Up to now, 170 species (105 dicots, 65 monocots) have been confirmed to be resistant to ALS inhibiting herbicides (Heap, 2022).

Two groups of mechanisms responsible of resistance to herbicides has been described; Non-Target Site Resistance and Target-Site Resistance (NTSR and TSR, respectively) (Gaines et al., 2020). Regarding ALS resistance, the TSR mechanisms are frequent, especially in broadleaf weeds (Yu and Powles, 2014a; Tranel et al., 2022). On the other hand, NTSR mechanisms can reduce (decreased foliar uptake, translocation, vacuolar sequestration or enhanced metabolism) the toxic form of the herbicide before reaching the target site. (Yu and Powles, 2014b; Hatami et al., 2016; Rigon et al., 2020; Torra et al., 2021a; Torra et al., 2021b). Of these, enhanced metabolism is one of the most important mechanisms; this may involve the enhanced activity of cytochrome P450 monooxygenases (P450s), glutathione-*S*-transferases (GSTs), glucosyltransferases, and ATP-binding cassette transporters (De Prado et al., 2005; Yasuor et al., 2010; Gaines et al., 2020; Palma-Bautista et al., 2020; Dimaano and Iwakami, 2021). Several cases of weeds resistant to ALS-inhibitor herbicides have been confirmed with NTSR mechanisms (mainly metabolism) (Yu and Powles, 2014b; Mei et al., 2017; Wang et al., 2019; Jugulam and Shyam, 2019; Han et al., 2021; Pan et al., 2022; Cao et al., 2022a; Shen et al., 2022; Feng et al., 2022; Lan et al., 2022; Zhao et al., 2022). Enhanced metabolism participates in grassweeds resistant to ALS- inhibiting herbicides, being rarely documented in dicots weeds (Hada et al., 2021). TSR involves point mutations in genes that encode specific herbicide target proteins or over expression of those genes Yu and Powles, 2014a; Iwakami et al., 2017; Gaines et al., 2020; Panozzo et al., 2021). Nowadays, over two dozen weed species have shown TSR mechanisms related with ALS-inhibitors resistance principally changes in nine amino acid positions of the *ALS* gene (Ala122, Pro197, Ala205, Phe206, Asp376, Arg377, Trp574, Ser653, and Gly654). Different amino acid changes lead to different levels of resistance (Tranel et al., 2022). It is known that Ala-122-Thr, Ala-205-Val, or Ser- 653-Asn/Thr amino acid changes in ALS usually causes resistance to IMI herbicides. In addition, changes in Pro-197 position grants high resistance to SU herbicides. Asp-376-Glu, Ala-205-Phe or Trp-574-Leu changes in ALS will provide broad spectrum resistance to all different chemical groups included in ALS-inhibiting herbicides (Cao et al., 2022b; Tranel et al., 2022). On the other hand, just one case of resistance was confirmed with Phe206Leu amino acid change in *Echinochloa crus-galli*; this mutation can give resistance to penoxsulam (TP), flucetosulfuron (SU) and flucarbazone-Na (SCT) (Fang et al., 2022).

So far, only two species of *Sinapis* spp. resistant to ALS inhibitors have been described worldwide: *S. arvensis*, which has been detected in seven countries (Australia, Canada, Iran, Italy, Spain, Turkey, and United States) and *S. alba*, only detected in two European countries (Cyprus and Spain). In *S. alba*, resistance to TM is due to a single amino-acid substitution of proline by serine at position 197 in the *ALS* gene; conversely, in *S. arvensis* several mutations have been found in the *ALS* gene: Thr122, Ser197, Glu376 and Leu574, (Warwick et al., 2005; Christoffers et al., 2006; Cruz-Hipolito et al., 2013; Gherekhloo et al., 2018; Khaledi et al., 2019; Sin and Kadioglu, 2021).

In the mountainous areas of southern Spain, the soils are sandy-loam and shallow. Winters have frequent rainfall and low temperatures (about 1 °C); however, temperatures are medium-high in summer (between 20 to 35 °C) and rainfall is infrequent. With these characteristics, cereal cultivation is the only possible annual crop for the farmers in the area The crop rotation is almost always the same winter wheat x barley x avena sativa, usually sown in late October and harvested in June of the following year. For more than 20 years, the use of TM has been the most widely applied herbicide in January for the control of dicotyledonous weeds. The selection pressure exerted by TM has ended the evolution of a resistant population of *S. alba* confirmed in 2008 in Malaga (southwest of Spain) due to TSR (Cruz-Hipolito et al, 2013). Ten years later, in 2018, farmers of an extensive area of ​​the province of Granada (southeast of Spain) found populations of *S. alba* not controlled by TM. The objectives were: (1) to determine the level of resistance to TM of three suspected-resistant populations of *S. alba* (R1, R2 and R3) collected from winter cereal fields in the southeast province; (2) to characterize the NTSR and TSR mechanisms involved in the resistance to this herbicide; and (3) evaluate the resistance profile to different herbicides with alternative modes of action (MOAs). Together, these data were analyzed to infer whether the *S. alba* resistant populations now present in two different regions of southern Spain (separated by a distance of 100 km) come from a common founder event or local evolution of resistance to TM has happened.

## 2 Materials and methods

### 2.1 Chemicals

For the whole plant greenhouse trials, the herbicides used were those listed in **Table 1**. For metabolism and enzyme activity assays (^14^C-tribenuron-methyl, 1660 kBq), technical grade TM as well as the TM (methyl 2-[[(4-methoxy-6-methyl-1,3,5-triazin-2-yl)-methylcarbamoyl]sulfamoyl]benzoate) metabolites metsulfuron-methyl (MM) (methyl 2-[4-methoxy-6-methyl-1,3,5-triazin-2-yl-carbamoylsulfamoyl]-benzoate) and hydroxylatedmetsulfuron-methyl (OH-MM) (methyl 2-[4-methoxy-6-methyl-1,3,5-triazin-2- y l- carbamoylsulfamoyl]-4-hydroxybenzoate) were obtained from DuPont de Nemours & Co (Nambsheim, France).

**Table 1:** Main characteristics of the herbicides used in this study to control Sinapis alba.

Herbicide	Company	Commercial Product	MOA (HRAC)	Field Dose (cc/g ai ha^-1^)
**Tribenuron-methyl**	Nufarm	75% w/v, Primma^®^ SL	ALS inhibitor/2	20
**2,4-D**	Nufarm	60% w/v, U 46 D Complet^®^ SL	Auxin mimics/4	600
**Clopyralid**	Corteva	72% w/v, Lontrel^®^ WG	Auxin mimics/4	300
**Dicamba**	Syngenta	48% w/v, Banvel^®^ D WG	Auxin mimics/4	144
**MCPA**	Nufarm	40% w/v, Procer M-40^®^	Auxin mimics/4	800
**Glyphosate**	Bayer CropScience	36% w/v, Roundup^®^ SL	EPSPS inhibitor/9	1080
**Metribuzine**	Bayer CropScience	60% w/v, Sencor^®^ SC	PS II inhibitor (serine 264 binder)/5	60
**Mesotrione**	FMC	10 w/v, Border^®^ SC	HPPD inibitor/27	150
**Pyraflufen-ethyl**	Belchim	2.65% w/v, Gozai^®^ EC	PPO inhibitor/14	7
**Flufenacet**	Belchim	60% w/v, Glosset 600 SC	VLCFAs inhibitor/15	240

### 2.2 Plant material

Seeds of *S. alba* plants with suspected resistance to TM (Rs= R1, R2 and R3) were harvested from cereal plots with rotation of winter wheat/barley/*Avena sativa* located in the north of the province of Granada. In addition, it was used a control population obtained from a field close to those plots where no herbicides were applied (**Table 2**). Seeds previously scarified manually were germinated in Petri dishes with filter paper moistened with distilled water and placed in a growth chamber. The samples were maintained at 28/18° C (day/night) with a photoperiod of 16 h, 300 μmol m^-2^s^-1^ flux of photosynthetic photons and 80% relative humidity. The seedlings were transplanted into pots containing sand: peat in a 1:1 (v/v) ratio and were placed in a greenhouse at 28/18° C (day/night) with a photoperiod of 16 h.

**Table 2:** Geographical situation of the *S. alba* populations, crop, history of herbicides and years of application.

Population	Crop	Herbicides	Years of application	Coordinates
**S**	No crop	No	0	37°03’58.7”N 3°50’00.3”W
**R1**	Cereals	TM/auxins	22/5	37°03’55.6”N 3°49’18.5”W
**R2**	Cereals	TM	12	37°03’48.9”N 3°49’34.4”W
**R3**	Cereals	TM/auxins	10/2	37°03’42.8”N 3°49’54.4”W

### 2.3 Whole plant assays

*Sinapis alba* plants with 3-4 leaves were treated with TM using a bench-type track sprayer (SBS-060 De Vries Manufacturing, Hollandale, MN, United States) equipped with 8002 flat fan nozzles and supplying 200 L ha^- 1^ to 250 KPa. TM was applied to: 0, 0.125, 0.25, 0.5, 1, 2, 4 and 10 g ai ha^-1^ for the susceptible (S) population and 0, 5, 10, 20 (field dose), 40, 80, 160, 320, and 640 g ai ha ^-1^ for the Rs populations. Ten plants were treated per dose, which were taken at randomized assay. Immediately after application, the plants were relocated in the greenhouse under the same ambient conditions described above. Four weeks after application (WAA), number of survival plants was recorded and cutted at ground level and dried at 60° C. After four days, samples were weighed, and data of dry weight and survival plant were transformed into a percentage relative to the untreated control to estimate the GR_50_ (dose required to reduce the weight of the shoots by 50% to the control) and LD_50_ (dose of herbicide required to kill a population of weeds by 50%) values. The experiment was repeated.

### 2.4 Non-target site resistance mechanisms study

#### 2.4.1 Absorption and translocation of TM

This trial aimed to discriminate the difference in absorption and translocation of TM in S and Rs *S. alba* plants. Rs and S plants were treated with TM solution prepared with ^14^C-herbicide mixed with the commercial formulation of the herbicide as the methodology described in Cruz-Hipolito et al. (2013). The final TM application rate was 20 g ai ha^−1^ in 200 L ha^−1^. The specific activity of the ^14^C-TM solution was 0.834 kBq μL^−1^. One drop (1 μL plant^−1^) of this mix-solution was applied to the adaxial surface of the leaf by using a microapplicator PB-600 (Hamilton Co., Reno NV, USA). All applied plants were kept under growing conditions described in previous section. Data of absorption and translocation of TM was obtained at 96 h after treatment (HAT) following the methodology described by Palma-Bautista et al., 2020. Tribenuron-methyl movement was performed on the three *Sinapis alba* populations. The application of ^14^C-TM was applied under the same conditions as in the previous assay. At 96 HAT plants were washed individually and pressed onto filter paper for two weeks. Visualization was performed using a store film (Storage Phosphor System) and a phosphor imager Cyclone (Perkin-Elmer, Packard BioScience BV, MA, USA.

#### 2.4.2 Metabolism of TM

A screening trial using a cytochrome p450 inhibitor (malathion) was performed on the three *S. alba* populations. Plants from S, R1, R2 and R3 were treated with malathion At 1000 g ai ha^-1^ and then dried for 1 h at room temperature (25 °C). Then TM was applied a field dose (20 g ia ha^-1^). Twenty days after, the fresh weight was obtained and analyzed to determine the reduction respect untreated control. Known that non-target site mechanism could be present, a metabolism assay of TM was carried out following the methodology described by Cruz-Hipolito et al. (2013). For that, young S and Rs plants were treated with mix of commercial TM and ^14^C-TM solution (20 g ai ha^−1^ in 200 L with a specific activity of 0.834 kBq μL^−1^). The application and recovery of the unabsorbed ^14^C-TM were made by using the same technique and washing solution was performed at 96 HAT. Metabolism was evaluated following the methodology described by and Gherekhloo et al. (2018). Chromatograms of the radioactive areas were obtained with a radioscanner (Berthold LB 2821, Wildbald, Germany) and each product was determined by comparison with known standards (tribenuron-methyl; TM, metsulfuron-methyl: MM, metsulfuronmethyl-hydroxylate: OH-MM and conjugate-MM). In this experiment, five plants from each population (Rs and S) were used and the experiment was repeated.

### 2.5 Target-site resistance mechanism study

#### 2.5.1 ALS enzyme activity

To study the inhibition of ALS activity by TM it was followed the methodology described by Cruz-Hipolito et al., 2013. ALS was extracted from 3 g of leaf tissue of S and Rs *S. alba* plants at the 6–8 leaf stage. Enzyme extract was used for measuring ALS activity at several doses of technical-grade TM (0, 0.1, 0.2, 0.4, 1, 2, 4 and 10 nM) for S and (0, 31,25, 62,5, 125, 250, 500, 1000 and 2000 nM) for Rs populations. The ALS enzyme activity was calculated by measuring acetoin production and expressing this as a percentage of control (no herbicide). The concentration of herbicide necessary to reduce ALS activity by 50% (I_50_) was calculated. The protein concentration of the crude extract was measured using Bradford (Bradford, 1976).

#### 2.5.2 *ALS* gene sequence

Three to four leaf-stage Rs and S populations of *S. alba* were treated with 20 g ia ha^-1^ of TM. Before the treatment, plant material was collected from 10 individual plants from each population and lyophilized. After a few days, all plants genomic DNA was extracted by using a plant DNA extraction kit (BIOTOOLS, B&M Labs S.A) following the manufacturer’s protocol. Three pairs of primers were used to amplify the conserved domain regions of the *ALS* gene (**Table 3**) spanning all the known mutation sites reportedly contributing to ALS resistance. The gene fragments of ALS were amplified from each individual and purified using the BIOTOOLS DNA Purification Kit. These purified gene fragments were sent to STAB VIDA (http://www.stabvida.com) to be sequenced with the primers listed above. The sequencing results were visualized and aligned using Geneious Prime software. Alignment was performed using as consensus sequence the GeneBank accession FJ655877 of *S. arvensis*.

**Table 3:** Pair of primers used in ALS gene sequence from four Sinapis alba populations.

Primer code	Sequence 5’- 3’	PCR conditions
ALS3B^a^	TCARTACTWAGTGCKACCATC	95°-5 min; 95°-30 sec, 54°-30 sec, 72°-1 min; 72°-10 min; 4° ∞
ALS3F	GGRGAAGCCATTCCTCC	
P1^a^	GAAGCCCTCGARCGTCAAGG	95°-5 min; 95°-30 sec, 60°-30 sec, 72°-1 min; 72°-10 min; 4° ∞
P2	CATAGGTTGWTCCCARTTAG	
SalF^b^	ATGAACTGGGGAGGTTTGTG	95°-5 min; 95°-30 sec, 60°-30 sec, 72°-1 min; 72°-10 min; 4° ∞
SalR	GCAAGGCATGAACAAGATTC	

^a^Designed by Cruz-Hipolito et al., 2013; ^b^Designed in this work.

### 2.6 Alternative chemical control

Several herbicides were used to study multiple resistance and find an alternative chemical control. For that, an experiment was carried out in the greenhouse over Rs and S *S. alba* populations (**Table 1**). Herbicides were applied using a laboratory chamber as was described above, at four-to-six leave stage. Four WAA, the number of surviving plants was counted, in addition, the plants were cut at ground level and weight to obtain the fresh weight (fw). The experiment had a random design with 10 plants (one plant per pot) for each herbicide dose. The experiments were carried out twice (spring and fall).

### 2.7 Statistical analysis

Regarding data from weight reduction, plant survival, and ALS enzyme activity were subjected to a three-parameter non-linear regression analysis. Thus, the dose of TM needed to reduce the dry weight (GR_50_), cause mortality (LD^50^), and inhibit the ALS activity (I_50_) by 50% were estimated. The log-logistic non-linear regression equation (1) used was the following:

y = d/[1 + b(log(x) – log(e))] (1)

Where d is the upper asymptote, b is the slope at the inflection point of the curve, and e is the herbicide rate at the inflection point; and x is the TM dose. The regression analyses were conducted using the package drc for the statistical environment (Ritz and Streibig, 2005). Resistance factors (RF)were computed as Rs/S GR_50_, LD_50_, or I_50_ ratios.

Data concerning the uptake, translocation, and metabolism assays were submitted to an analysis of variance (ANOVA) using the Statistix (version 10.0) (Analytical software, USA) software. The requirement of homogeneity of variance was verified with the Bartlett test, and the normality of the data was analyzed with the Shapiro–Wilk test. Differences with p < 0.05 were considered significant and a Tukey’s test was conducted to compare the means as necessary.

## 3 Results

### 3.1 Whole plant assays

The TM dose required to control the Rs *S. alba* population by 50% (LD_50_) was higher than the field recommended dose (20 g ai ha^-1^) used in cereal crops in Spain, while for the S population this dose was below the field dose (data not shown). Based on the GR_50_ values, the R1, R2 and R3 populations were 57.4, 46.5 and 57.0 times more resistant than the s population, respectively (**Table 4**).

**Table 4:** Parameters of the log-logistic used to estimate dose-response curves (% dry weight) in populations of *S. alba* (S) and R to TM.

Population	d	d	GR_50_	*p*-value	RF
**S**	99.9	0.9	0.34 ± 0.05	<0.001	–
**R1**	101.9	1.5	19.5 ± 2.3	<0.001	57.4
**R2**	101.5	1.3	15.8 ± 1.3	<0.001	46.5
**R3**	101.9	1.4	19.4 ± 2.1	<0.001	57.0

### 3.2 Absorption and translocation of TM

**Figure 1:**
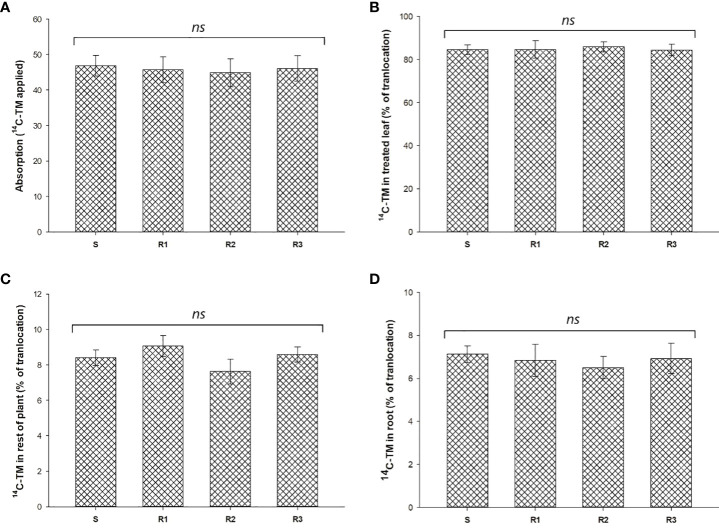
Percentage of ^14^C-TM absorption **(A)** and translocation **(B–D)** in resistant (R1, R2 and R3) and susceptible (S) populations of *S. alba*. Vertical bars represent ± standard errors of the mean (n = 5x 2). ns, not significant difference.

**Figure 2:**
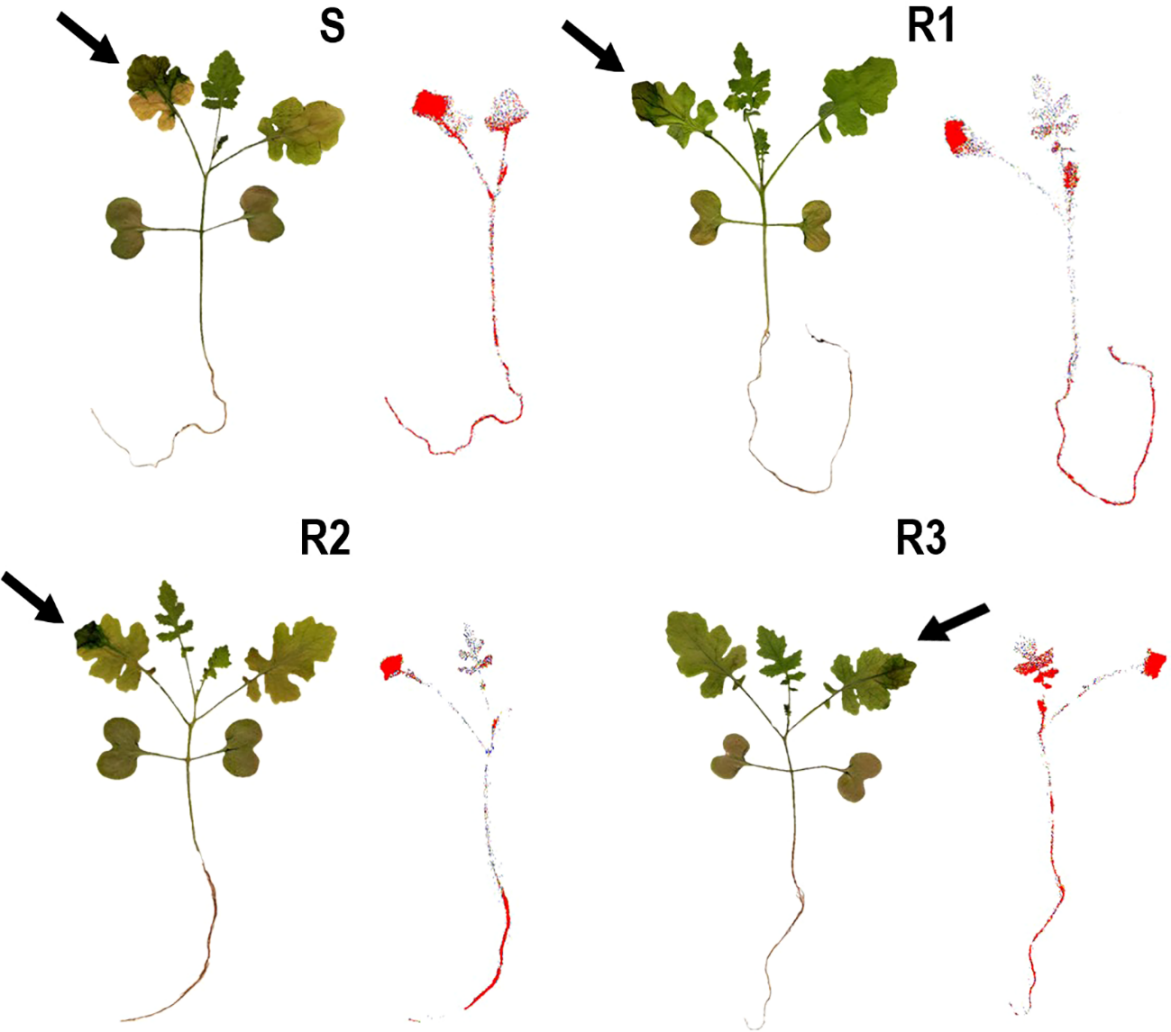
Digital images and ^14^C-tribenuron-methyl visualization in *Sinapis alba* populations (S, R1, R2 and R3). Arrows indicate the treated leaves. The concentration of ^14^C-tribenuron-methyl is highlighted in red.

^14^C-TM recovered in *S. alba* plants was from 94.2 to 96.3% (data not shown). TM absorption was close to 50% at 96 HAT and no significant differences were found between populations (**Figure 1A**). The movement of ^14^C-TM from the treated leaves to the shoots and the root system was about 15% in all the populations studied. No significant differences were observed between the Rs and S populations 96 HAT (**Figures 1B-D**, and **Figure 2**).

### 3.3 Metabolism of TM

**Figure 3:**
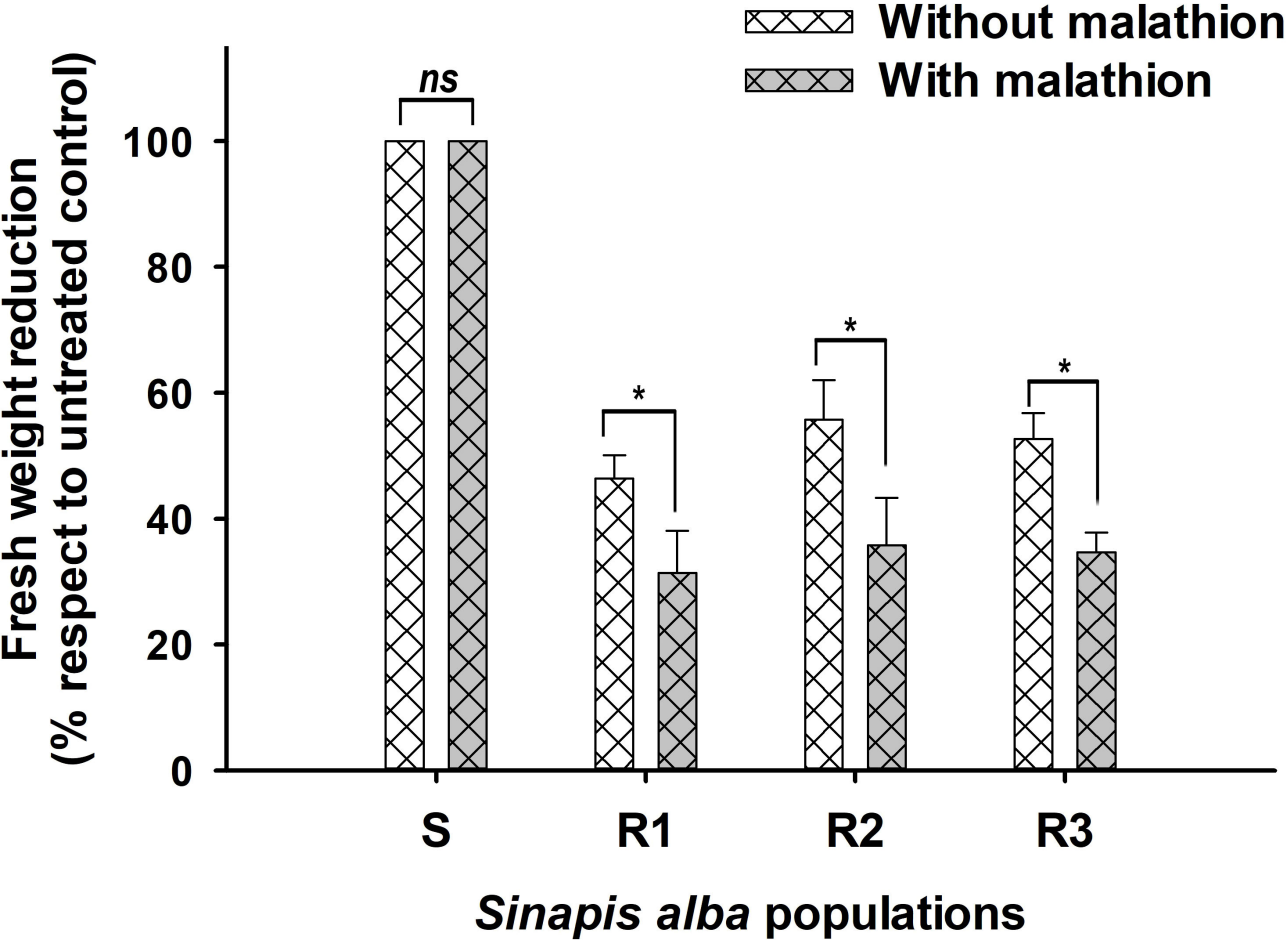
Fresh weigh reduction of four *Sinapis alba* populations treated with herbicide only (without malathion) and with malathion (1000 g ia ha^-1^) plus herbicide. * Significant difference (n=20). ns, non significant difference.

**Figure 4:**
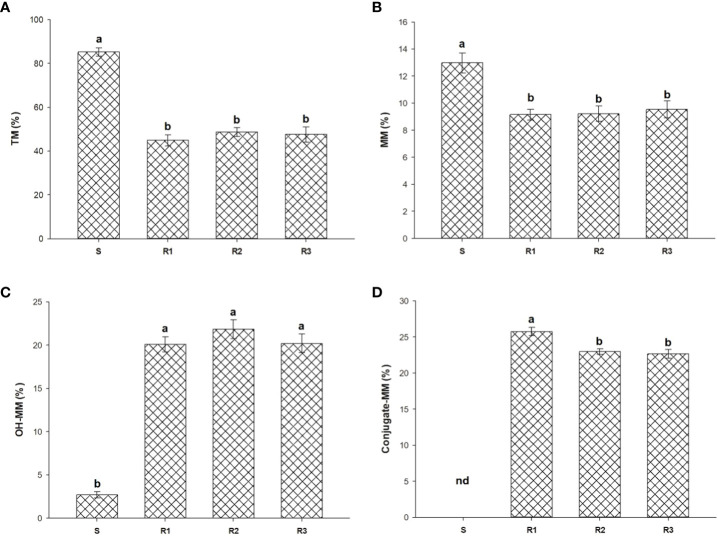
^14^C-TM **(A)** and leaf extracted metabolites **(B–D)** by TLC at 96 HAT in R1, R2, R3 and S *S. alba* plants treated with 20 g ai ha^-1^. Vertical bars represent ± standard errors of the mean (n = 5). nd, not detected. Different letters on bars represents the Tukey´s group (α = 0.05) after mean comparison.

Screening using malathion as Cyt.P450 inhibitor shown a slight reduction of fresh weigh in R populations. As shown in **Figure 3**, R populations had differences when were exposed to TM and TM plus malathion. The amount of ^14^C-TM detected in the S was higher than that found in the Rs plants at 96 HAT (85.2%). However, in Rs populations, three metabolites (MM, OH-MM, and conjugated-MM) were detected (**Figure 4**) at that time. N-demethylation of TM to form MM (active sulfonylurea herbicide) was the minor metabolite formed and represented only 9.2 to 13.0% of applied ^14^C-TM in all populations studied. The TM and MM metabolites were further degraded, mainly through hydroxylation (Cyt.P450 monooxygenase system) of the phenyl ring to OH-MM. The amount of OH-MM in the populations R1, R2 and R3 were 7.4, 8.1 and 7.5 higher than in the S plant population, respectively (**Figure 2**). Finally, the third metabolite, the MM-conjugate, was formed by conjugation of OH-MM with carbohydrates. The levels of MM-conjugate were very high in the Rs populations but null in the case of the S populations.

### 3.4 ALS enzyme activity

The specific ALS activity showed no differences between Rs and S *S. alba* plant populations (**Table 5**). Based on the concentrations of the TM needed to reduce the ALS activity by 50% (I_50_) the S (0.32nM) population was considerably more sensitive than the Rs populations 340.9, 330.6 and 345.1 for R1, R2 and R3, respectively). (**Table 5**).

**Table 5:** Parameters log-logistic used to estimate the concentration of TM necessary to reduce the activity of the ALS enzyme by 50% (I_50_) in *S. alba* populations.

Population	ALS^a^ activity	d	b	I_50_ (nM)	*p*-value	RF
**S**	355.3 ± 5.3	98.7	1.7	0.32 ± 0.01	<0.001	–
**R1**	364.5 ± 2.5	99.5	1.8	340.9 ± 15.1	<0.001	1062.5
**R2**	359.4 ± 6.9	99.4	1.7	330.6 ± 19.1	<0.001	1033.1
**R3**	361.8 ± 7.2	99.2	1.8	345.1 ± 25.6	<0.001	1078.4

^a^nmol of acetoin mg^-1^ of protein h^-1^.

### 3.5 ALS gene sequence

**Figure 5:**
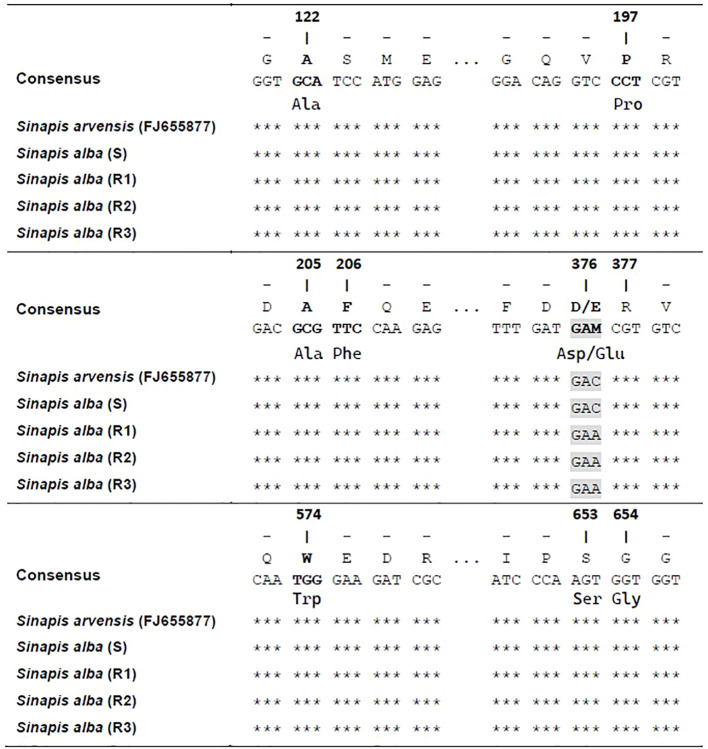
Alignment of ALS gene sequence of plants from S and Rs populations of *S. alba* compared to a S population of *Sinapis arvensis* (FJ655877). Positions refer to the known ALS gene sequence of Arabidopsis thaliana (X51514). *** refers to similarity with respect to individual nucleotides in the consensus.

Three fragments of the ALS gene were amplified from each of the S and Rs plants and compared with that of *S. arvensis* (GenBank, accession FJ655877). Several single-nucleotide polymorphisms were identified, but they did not result in any amino acid substitutions. On the other hand, a single-nucleotide change occurred in aminoacid position 376 (GAC to GAA) in the three resistant *S. alba* populations (**Figure 5**), giving the amino acid change Asp-Glu.

### 3.6 Alternative chemical control

Based on the results obtained, we can classify the effect of the different herbicides tested into three groups: (1) the herbicides 2,4-D and MCPA (auxin mimics, phenoxy-carboxylic-acids), as well as the herbicides glyphosate (EPSPS inhibitor), metribuzine (PS II-Serine 264 Binders**)** and mesotrione (HPPD inhibitor) with great control efficacies against the plants producing total mortality (2) the herbicides pyraflufen (PPO inhibitor) and flufenacet (VLCFAS inhibitor) that show phytotoxic effects (loss of pigments) during the first week but, later on, the plants recovered and showed survival percentages ranging from 40 (pyraflufen) to 100% (flufenacet), and (3) herbicides clopyralid (auxin mimics, pyridine carboxylic acids) and dicamba (auxin mimics, benzoic acids) that showed low control over the S and Rs populations of *S. alba* included in this study (**Table 6**).

**Table 6:** Effect of alternative herbicides at recommended field dose on *S. alba* populations fresh weight (fw) and survival (%) plants populations.

Herbicide	S1		R1		R2		R3	
	fw^a^	Survival (%)	fw	Survival (%)	fw	Survival (%)	fw	Survival (%)
**Control**	4.3 ± 0.3	100	4.6 ± 0.4	100	4.1 ± 0.8	100	4.3 ± 0.2	100
**2,4-D**	0.0	0	0.0	0	0.0	0	0.0	0
**Clopyralide**	3.4 ± 0.4	100	4.1 ± 0.3	100	3.4 ± 0.4	100	4.1 ± 0.4	100
**Dicamba**	2.8 ± 0.6	100	3.2 ± 0.3	100	3.1 ± 0.3	100	3.4 ± 0.3	100
**MCPA**	0.0	0	0.0	0	0.0	0	0.0	0
**Piraflufen**	1.2 ± 0.6	60	0.8 ± 0.8	50	0,4 ± 0,1	40	0.7 ± 0.6	50
**Glyphosate**	0.0	0	0.0	0	0.0	0	0.0	0
**Metribuzine**	0.0	0	0.0	0	0.0	0	0.0	0
**Mesotrione**	0.0	0	0.0	0	0.0	0	0.0	0
**Flufenacet**	3.0 ± 0.7	100	2.8 ± 0.9	100	2.6 ± 0.6	100	2.8 ± 0.9	100

^a^fw: fresh weight (n=20).

## 4 Discussion

*Sinapis alba* is an invasive broadleaf severely infesting wheat fields in southwest Spain. The use of TM is one of main tools to control dicotyledonous weeds in wheat. However, the continued use of this herbicide has led to the emergence of resistant biotypes of *Sinapis alba* since 2008. (Rosario et al., 2011; Cruz-Hipolito et al., 2013). Here we included three *S. alba* populations found in 2018 that were not controlled by TM in Granada (southeast Spain, separated by more than 100 km that the one found in 2008). They were collected from a cereal system with a winter wheat/barley/*Avena sativa* rotation. The local farmers highlighted that the area where the Rs populations were collected had been mainly treated with TM for 10 (R3), 12 (R2) and 22 (R1) consecutive years.

In this study, we demonstrated that these resistant populations developed high-level resistance to TM with a R/S GR_50_ ratio of 57.4, 46.5 and 57.0 (R1, R2 and R3, respectively). These values were higher than those found for *S. alba* in Spain Rosario et al., 2011; Cruz-Hipolito et al., 2013). The alignment of sequences from the *S. alba* populations included in this study showed an Asp376Glu substitution in the *ALS* gene. The Asp376Glu substitution has been found in other weed species: *Amaranthus* spp., *Kochia scoparia*, *Monochoria vaginalis*, *Conyza canadensis*, *Galium* spp. *Raphanus raphanistrum*, *Descurainia sophia* and *Lolium perenne* (Yang et al., 2018; Tranel et al., 2022; Palmieri et al., 2022). Former studies have shown that ALS mutations at Asp376 positions commonly confer broad-spectrum resistance to many ALS inhibitors (Zakaria et al., 2021; Tranel et al., 2022). Moreover, it has been confirmed that Asp376Glu conferred higher resistance values to TM than other mutations in the ALS gene in other weed species (Zhang et al., 2017). Previous studies on *S. alba* and *S. arvensis* resistant to ALS inhibitors have shown that the main mechanism responsible for that resistance was different mutations in the ALS gene, at several positions Pro197, Trp574 and Ala 122 (Warwick et al., 2005; Rosario et al., 2011; Cruz-Hipolito et al., 2013; Khaledi et al., 2019; Sin and Kadioglu, 2021). There is just one case with the change Asp376Gly in a biotype of *S. arvensis* (Khaledi et al., 2019), and another one with the Asp376Glu change in *S. arvensis* as well (Bahmani et al., 2015). This work demonstrates the occurrence of the Asp376Glu change in *S. alba* for the first time worldwide.

Other resistance mechanisms, such as lower herbicide absorption or translocation, excluded in Rs populations. However, metabolism studies showed that TM is metabolized faster to non-toxic compounds (OH-MM and MM-conjugate) in resistant populations. OH-MM is formed thanks to the activity of P450 while MM-conjugate thanks to GS, in a well-known degradation route for TM in broadleaf weeds (Cruz-Hipólito et al., 2013; Palma-Bautista et al., 2022). It has been shown in recent years that cases of resistance due to metabolism are increasing mainly in grass weeds, as specified in the introduction section. To date, metabolism-based resistance to ALS inhibitors has been identified in few dicotyledonous weeds, including *S. arvensis*, *Amaranthus tuberculatus*, *Papaver rhoeas* and *Descurainia sophia* (Veldhuis et al., 2000; Liu et al., 2018; Hada et al., 2021; Torra et al., 2021b; Xu et al., 2022). Studies on metabolic herbicide resistance in broadleaf weed species are restricted, with genes associated with herbicide metabolism almost remaining unknown. Shen et al., 2022 demonstrated that *cyt P450* gen (CYP77B34) was responsible for TM metabolism in *D. sophia*, also a Brassicaceae weed species.

The non-target site resistance mechanism based on enhanced metabolism is a dramatic scenario in terms of weed control. There is a possibility that this metabolism may be involved in resistance to different MoAs, even if the weed has never been exposed to those herbicides (Gaines et al., 2020; Torra et al., 2021a). In our studies, several herbicides with different MOAs, 2,4-D and MCPA (auxin mimics), glyphosate (EPSPS inhibitor), metribuzin (PS II inhibitor Serine 264 binder)) and mesotrione (HPPD inhibitors) could control these Rs populations. The potential detoxification enzyme pathway may have high catalytic efficiency and strong substrate specificity (Shen et al., 2022). However, our work needs further research focused on finding molecular genes involved in the high expression of metabolism.

The coexistence of more than one resistance mechanism within the same species, population or individual can happen. For example, TSR and NTSR (enhanced metabolism) mechanisms to ALS-inhibiting herbicides were found in single *P. rhoeas* and *Setaria viridis* plants (Rey-Caballero et al., 2017; Huang et al., 2021). The presence of different resistance mechanisms together may be due to combination through cross-pollination. Weed species with high levels of cross-pollination are more likely to accumulate diverse resistance mechanisms (to a single herbicide or to multiple herbicides), and for this process to occur more rapidly than in self-pollinated species (Gaines et al., 2020). In the resistant population of *S. alba* found in Malaga in 2008, the main mechanism of resistance was a mutation in the *ALS* gene. In those Rs populations found in Granada (100 km apart from Malaga) both TSR (Glu376 in *ALS* gene) and NTSR (enhanced metabolism) mechanism are involved in the resistance to ALS inhibitors. From an evolutionary biological perspective, enhanced herbicide metabolism can be recognized as a generalist adaptive response, which can combine with other mechanisms including TSR, a specialist adaption, to confer higher levels of resistance (Comont et al., 2020; Xu et al., 2022). Our study is the first report worldwide of NTSR to TM in *S. alba* and also the first report of above-mentioned amino acid change in this species. NTSR to ALS inhibitors had already frequently been reported in grass weeds, but rarely in dicotyledonous weeds, as previously stated. Here, we observed that NTSR together with TSR mechanism can confer higher resistance levels in the *S. alba* Rs populations included in this study than in those one found in Malaga in 2008. Our results suggest that the mechanism of resistance involved to TM is at least in part different and that independent events of evolution of TSR and/or NTSR occurred across south Spain in *S. alba* populations under high selection pressure with ALS-inhibiting herbicides. Our results also suggest that once an R haplotype has evolved, it is geographically spread in a region (we have three suspected populations collected from three different fields in Granada). Wind-borne pollen and/or seeds movement by human activity (mainly machinery shared between fields) could a vector for herbicide resistance alleles in these populations as occurs for other weed species (Loubet et al., 2021). This last factor might be involved in the long-distance dispersal of *S alba* between Granada and Malaga, something that did not seem to be the case, according to the resistance mechanisms found in both regions.

Our results provide a different and better understanding of TM resistance in *S. alba*, which helps design effective strategies for weed management. The ALS gene mutation found here could give cross-resistance to different ALS-inhibiting herbicides. Whole plant trials would be needed to corroborate this hypothesis. Moreover, the R *S. alba* populations can be effectively controlled with several herbicides with different MOAs. Nine different herbicides, belonging to six different alternative MOAs were tested at field recommended doses against the Rs populations. As stressed in the results section, while auxin mimics (excepting clopyralid and dicamba), EPSPS, HPPD and PS II inhibitors rendered maximum control levels, PPO and VLCFAS did not achieve acceptable efficacies. Therefore, there are available enough MOAs to be considered in rotation or mixtures when designing integrated weed management programs for *S. alba*, though the best would be a rotation of mixtures, to avoid or delay resistance onset (Busi et al., 2020). For example, previous studies showed that properly using auxin mimics, PS II and ALS inhibitors against broadleaf weeds, resistance evolution to ALS inhibitors can be completely mitigated (Beckie and Reboud, 2009).

Furthermore, other weed control methods, rather than relying on chemical control, must be implemented on long-term weed management programs to avoid the evolution of different resistance mechanisms.

## Data availability statement

The data presented in the study are deposited in the GenBank repository, accession number OP681621 (biotype R) and OP681622 (biotype S).

## Author contributions

Conceptualization, RP.; methodology, MO, JV-G, CP-B, JT.; investigation, BG, J-VG, CP-B, JP; data curation, MO, J-VG, CP-B, JT; writing—original draft preparation, RP, J-VG, MO; writing—review and editing, RP, J-VG, MO, CP-B, JT, JP.; supervision, MD, RP. All authors have read and agreed to the published version of the manuscript.

## Funding

This work has been supported by the grant GR from the Government of Extremadura and the Asociacion de Agroquimicos y Medio Ambiente (Spain). JT acknowledges support from the Spanish Ministry of Science, Innovation, and Universities (grant Ramon y Cajal RYC2018-023866-I).

## Acknowledgments

We are grateful to Estefania Minero for technical help.

## Conflict of interest

The authors declare that the research was conducted in the absence of any commercial or financial relationships that could be construed as a potential conflict of interest.

## Publisher’s note

All claims expressed in this article are solely those of the authors and do not necessarily represent those of their affiliated organizations, or those of the publisher, the editors and the reviewers. Any product that may be evaluated in this article, or claim that may be made by its manufacturer, is not guaranteed or endorsed by the publisher.
